# Southern African HIV Clinicians Society 2023 Guideline for post-exposure prophylaxis: Updated recommendations

**DOI:** 10.4102/sajhivmed.v24i1.1522

**Published:** 2023-09-28

**Authors:** Jaco Horak, Willem D.F. Venter, Camilla Wattrus, Nectarios Papavarnavas, Pauline Howell, Gillian Sorour, Carole Wallis, Katherine Gill, Francesca Conradie, Linda-Gail Bekker

**Affiliations:** 1 The Desmond Tutu HIV Centre, University of Cape Town, Cape Town, South Africa; 2 Ezintsha, Faculty of Health Sciences, University of the Witwatersrand, Johannesburg, South Africa; 3 Southern African HIV Clinicians Society (SAHCS), Johannesburg, South Africa; 4 Department of Medicine, Division of Infectious Diseases and HIV Medicine, University of Cape Town, Cape Town, South Africa; 5 Clinical HIV Research Unit, University of the Witwatersrand, Johannesburg, South Africa; 6 Department of Paediatrics and Child Health, University of the Witwatersrand, Johannesburg, South Africa; 7 BARC-SA and Lancet Laboratories, Johannesburg, South Africa; 8 Clinical HIV Research Unit, Wits Health Consortium, Johannesburg, South Africa; 9 Department of Medicine, University of the Witwatersrand, Johannesburg, South Africa; 10 Helen Joseph Hospital, Johannesburg, South Africa

## Contents

1. Introduction

2. A summary of PEP administration

 2.1. Immediate services

 2.2. Additional considerations

3. A stepwise approach to providing PEP

 3.1. Step 1: Prevention of HIV exposure

 3.2. Step 2: Initial assessment following exposure

 3.3. Step 3: HIV transmission risk assessment

  3.3.1. Risk of transmission in an occupational setting

  3.3.2. Non-occupational and sexual exposures

 3.4. Step 4: Assessing the source individual

 3.5. Step 5: Assessment of the exposed individual

  3.5.1. HIV testing

 3.6. Step 6: Initiating PEP in the exposed individual

  3.6.1. Selecting an appropriate ARV regimen

  3.6.2. Counselling and support

  3.7. Step 7: Follow-up

4. Other considerations

 4.1. Hepatitis B

 4.2. Hepatitis C

 4.3. Sexually transmitted infections

 4.4. Pregnancy

 4.5. Tetanus

 4.6. Malaria

 Acknowledgements

 References

## Key summary points

The administration of a short course of antiretroviral therapy to an HIV-negative person who has been exposed to the human immunodeficiency virus (HIV-1) to prevent acquisition of the virus is known as post-exposure prophylaxis (PEP):

- PEP is used as a preventive intervention.
- PEP is an emergency intervention for *all* persons exposed to HIV.
- The exposure may be occupational or non-occupational.
- The route of exposure may be sexual, percutaneous or via non-genital mucosal membranes.
- PEP can effectively prevent infection in a person exposed to HIV when initiated as soon as possible and at least within 72 h post-exposure.
- While one or two antiretrovirals can be used in pre-exposure prophylaxis (PrEP), the global recommendation for PEP is a 3-drug regimen involving, whenever possible, an integrase inhibitor (usually dolutegravir).
- The risk:benefit assessment falls firmly in favour of prescribing PEP, as integrase inhibitor-based regimens are very safe.
- The recommended duration of a course of PEP is 28 days. The full 28-day course should be provided at the time of PEP initiation.
- Almost all PEP is now 28 days of first-line ART (i.e., tenofovir disoproxil, lamivudine and dolutegravir [TLD]).
- In instances of repeated PEP use in an individual, it is important to assess the potential benefit of PrEP going forward.

## 1. Introduction

South Africa has the largest number of people living with HIV (PLWH) of any country in the world. The Joint United Nations Programme on HIV/AIDS (UNAIDS) estimates the prevalence in South Africa is ~8 million with an incidence of ~7 per 1000 among 15–49-year-olds.^1^ Despite improved roll-out of antiretroviral treatment (ART), modelling studies have shown the incidence of HIV among young women and girls has remained high in southern Africa.^2,3^ Almost a quarter of women of reproductive age (15–49 years) are living with HIV. The vertical transmission rate in the country is 3.5%, with many new infections occurring among young women during late pregnancy.^4^ In addition, South Africa’s high rate of sexual violence means that these individuals may be exposed to a high risk of HIV transmission due to sexual transmission risk and sexual violence-related injury. Half of new infections occur among young key populations – people who have a high vulnerability to HIV due to their behaviours and potential sexual violence and who also experience barriers to effective prevention services. These include men who have sex with men (MSM), transgender women (TGW), people who sell sex, or people who inject drugs (PWID). Therefore, it is vital that these populations have access to effective HIV prevention.

Vital to curbing the rate of new HIV infections is effective prevention, with pre- and post-exposure prophylaxis playing a role. Currently, the World Health Organization recommends pre-exposure prophylaxis (PrEP) based on clinical trials in MSM and TGW, discordant couples and heterosexual populations,^5,6,7,8^ and South African PrEP guidance has been published.^9^ Post-exposure prophylaxis (PEP) has been in existence for decades, since it was shown in a case-control study of occupational exposures among healthcare workers that zidovudine reduced HIV acquisition by almost 80%.^10^ The risk of HIV transmission in a healthcare setting has been reported as 0.3% through percutaneous exposure to the blood of a source individual living with HIV^10^ and 0.09% after a mucous membrane exposure.^11^ With the use of potent antiretroviral (ARV) medications that have increased bioavailability, it was presumed the use of a 3-drug PEP regimen would reduce this risk further. In the current era of increasing viral suppression in individuals with HIV, early and appropriate PEP initiation, and improved infection control protocols, these rates may be even lower. A literature review conducted in 2016 documented 10 seroconversions among 7652 healthcare-related exposures.^12^

The risk may be significantly higher in cases of percutaneous exposure in which more than one risk factor is present (e.g., in individuals who incur a deep injury with a hollow-bore needle from an HIV-positive source individual who has a high viral load). Although the effect of viral load has not been studied in individuals with occupational exposures, there is evidence that the probability of sexually transmitting HIV is correlated with the source individual’s HIV viral load.^13,14,15,16^ Table 1 details estimated likelihood of HIV transmission according to different modes of exposure.^17^

**TABLE 1:** Estimated likelihood of HIV transmission according to different modes of exposure.

Mode of exposure	Likelihood of transmission	
	Per 10 000 exposures	CI
Receptive anal intercourse	138	102–186
Receptive penile-vaginal intercourse	8	6–11
Needle sharing injection	63	41–92
Percutaneous (healthcare workers)	23	0–46
Insertive anal sex	11	4–28
Insertive penile-vaginal intercourse	4	1–14

*Source*: Patel P, Borkowf CB, Brooks JT, Lasry A, Lansky A, Mermin J. Estimating per-act HIV transmission risk: A systematic review. AIDS (London, England). 2014;28(10):1509. https://doi.org/10.1097/QAD.0000000000000298

CI, confidence interval.

Awareness and use of PEP among populations that could benefit from it is universally low. In a 2022 systematic review of over 12 000 MSM, the pooled estimate of individuals who were aware of PEP was modest, at 59.9% (95% confidence interval [CI]: 50.5–68.7), and that of those who had previously used PEP was very low, at 4.9% (95% CI: 2.4–9.8).^18^ The study concludes that PEP is an underused HIV prevention strategy among MSM and that once individuals become aware of PEP, the majority are willing to use it if they are supported appropriately in terms of individual, social, and structural barriers. We know this ‘under use’ is also true among young women, both for unanticipated sexual exposure and sexual violence. With children, PEP may be used post-partum to prevent vertical transmission from a mother with HIV, and it may also be used in the case of blood product contamination or sexual violence.

Given significantly more HIV transmission occurring as a result of non-occupational exposures in southern Africa, and a lack of awareness among both potential beneficiaries and healthcare workers alike, this guideline has been updated to include PEP recommendations for *all* potential HIV exposures with an individualised risk assessment approach.

It is to be noted that this guideline does not cover PEP recommendations for vertical transmission, and only briefly covers PEP guidance for individuals < 10 years old or < 30 kg in weight. For comprehensive guidance for these individuals, consult the South African National 2023 Guideline for Vertical Transmission Prevention of Communicable Infections (2023),^19^ the South African National 2020 Guidelines on the Management of PEP in Occupational and Non-Occupational Exposures^20^ or Chapter 9 of the 2023 Paediatric Hospital Level Standard Treatment Guidelines and Essential Medicines List for South Africa.^21^

## 2. A summary of PEP administration

**FIGURE 1:**
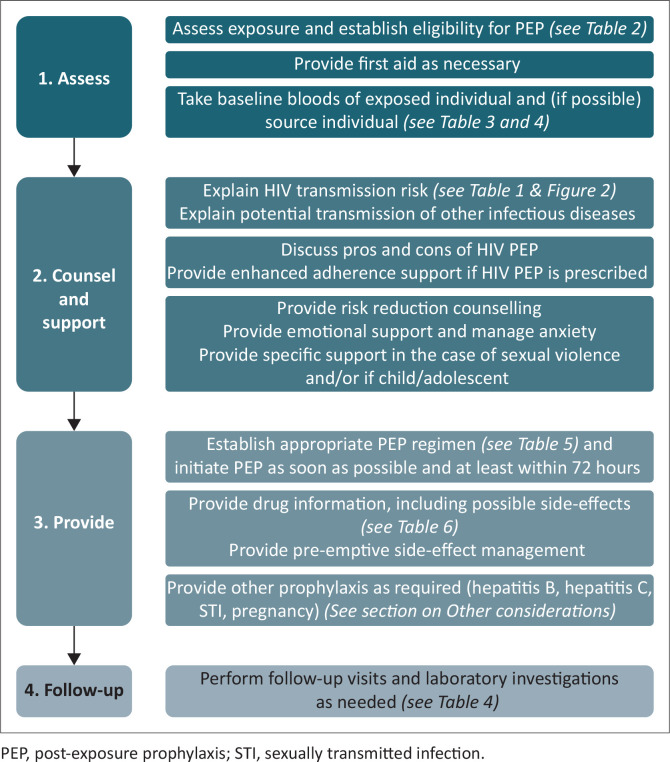
Summary of PEP administration.

Figure 1 summarises the recommended approach to providing PEP services. Administration requires the provision of immediate services along with additional considerations.

**FIGURE 2:**
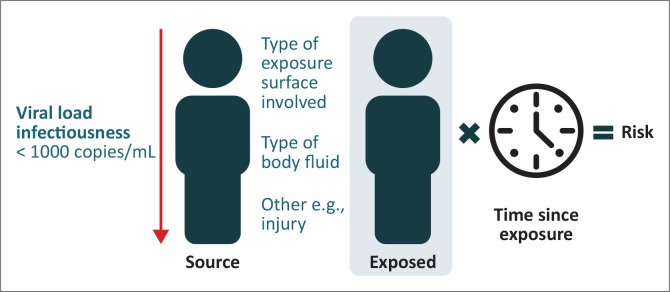
A summary of post-exposure risk.

### 2.1. Immediate services

- Provide first aid (if required)
- Establish PEP eligibility
- Provide immediate PEP single dose *as soon as possible*
- Perform HIV test on exposed individual and (if possible) source individual
- Provide emergency contraception (if required)
- Provide counselling and emotional support
- Establish appropriate PEP complete regimen and provide side-effect management.

### 2.2. Additional considerations

- Screen for and manage:
  - sexually transmitted infections (including syphilis)
  - pregnancy
  - hepatitis B and C
  - tetanus and other potentially transmissible infectious diseases (e.g., malaria) if contextually appropriate
  - gender-based/intimate partner violence.
  - Provide ongoing support and counselling.
  - Provide HIV counselling, prevention and treatment as necessary.

## 3. A stepwise approach to providing PEP

### 3.1. Step 1: Prevention of HIV exposure

Prevention of exposure to HIV remains the cornerstone in reducing the number of new HIV infections. There is now a growing selection of HIV prevention modalities and these need to be applied in different settings and times in an individual’s life course. HIV transmission can be prevented by use of barrier protection (latex condoms plus lubricant) during sexual encounters, the effective use of PrEP, undetectable viral loads in serodiscordant sexual partners living with HIV (Undetectable = Untransmittable; U=U), harm reduction practices in PWID and strict adherence to universal precautions within the healthcare setting. However, despite such precautions, potential HIV exposures do occur, and it is important in such situations that PEP be started as soon as possible (and no later than 72 h) following the exposure to ensure the smallest possible risk of HIV transmission. *All cases of potential exposure to HIV should be considered as a medical emergency.* Where exposures are frequent, it may be useful to discuss PrEP, but this can be done after managing the immediate exposure with PEP.^9^

### 3.2. Step 2: Initial assessment following exposure

It is important to understand what the definition of HIV exposure is. This refers to *any* person encountering infectious bodily fluids from someone (the source individual) that is known to be living with HIV or in whom the HIV status is unknown and carries a risk of HIV transmission to the exposed individual (i.e., a detectable viral load). Risk of transmission is extremely low (at least in sexual transmission) when the viral load in the source individual is < 1000 copies/mL and essentially untransmittable when the source viral load is undetectable (U=U).^22^ This has not been studied in non-sexual transmission. HIV exposures (excluding vertical transmission from mother to child) can occur in many different scenarios but can be broadly divided into three main categories, namely occupational (healthcare worker-associated), consensual sexual contact, and trauma/assault/non-consensual sexual exposure. *Though the assessment and examination may differ between these groups depending on the nature of the exposure, management in terms of HIV PEP therapy remains essentially the same.* Prevention of vertical transmission is covered elsewhere.^19,21^

Every person that reports an exposure to potentially infectious fluid should, if possible, be assessed by a healthcare provider. Essential components of the initial assessment include establishing the mechanism and nature of the exposure, the HIV status of the source individual and the infectiousness of the fluid that the person was exposed to, as well as examination of the exposed area. If appropriate and not already done, first aid should be provided, including thoroughly washing the injury with soap and water, or thoroughly irrigating the exposed mucous membranes.

In addition, in cases of occupational exposure or sexual assault, a detailed recorded history of the exposure is important for potential legal purposes. All healthcare providers who suspect or recognise child abuse should examine such cases thoroughly, meticulously collect evidence, accurately document the clinical findings, provide the necessary treatment (prophylactic and/or other), and refer the child for appropriate counselling. It is also a legal requirement to report such cases and the healthcare provider must be prepared to testify to their findings in a court of law. All children < 14 years of age with suspected or proven penetrative injuries should have specimens collected using the Paediatric Sexual Assault Evidence Collection Kit (SAECK) and a J88 form and Form 22 should be completed and handed over to the police within 24 h.^20^

In all individuals, possible transmission of other infections (including hepatitis B and C and sexually transmitted infections [STIs]) and possible risk of pregnancy should be considered. Recommended baseline and follow-up investigations are detailed in later steps.

*In instances of repeated PEP use, it is important to assess the potential benefit of PrEP going forward*. Recommendations for PrEP can be found elsewhere.^9^

### 3.3. Step 3: HIV transmission risk assessment

The risk of acquiring HIV following an exposure is determined by the nature of the exposure and the infectiousness of the source individual. High-risk exposures involve exposure to a larger quantity of virus from the source individual, either due to exposure to a larger quantity of infectious fluid (e.g., blood transfusion) or due to high amounts of circulating HIV (i.e., a high viral load) in the source.

It is important to remember that not all body fluids carry the same risk of HIV transmission. Familiarisation with the types of fluid that have the potential to transmit HIV (see Table 2) will greatly assist in the initial risk assessment and guide the decision as to whether PEP is required or not. It should be kept in mind that non-infectious fluids may be secondarily contaminated with infectious fluid (blood, etc.), and it is therefore crucial to get as much information as possible regarding the type of fluid the person was exposed to. In some scenarios this may be impossible to determine accurately, but the decision should be to err on the side of caution and have a lower threshold to prescribe PEP if unsure.

**TABLE 2:** Infectious and non-infectious fluids.

Potentially infectious	Non-infectious (in the absence of contamination by an infectious fluid)
Blood, including any blood-stained fluid or tissue	Saliva
Vaginal secretions	Sputum
Penile pre-ejaculate and semen	Tears
Rectal fluid	Sweat
Tissue and wound fluid	Urine
Any fluid drained from a body cavity, including cerebrospinal, ascitic/peritoneal, pleural, pericardial, synovial and amniotic fluid	Stool
Breast milk	

#### 3.3.1. Risk of transmission in an occupational setting

An increased risk of HIV transmission is associated with:

- A deep percutaneous sharp injury
- Percutaneous exposure involving a hollow needle that was used in a vein or artery
- Visible blood on the sharp instrument involved in a percutaneous injury
- When the source individual has a detectable HIV viral load.

Post-exposure prophylaxis is not indicated in:

- Cases where the source individual is known to be HIV-negative and unlikely to be in the window period
- Exposure to non-infectious fluid only
- Exposure to infectious fluid on intact skin only
- Cases where the exposed individual is known to be living with HIV.

#### 3.3.2. Non-occupational and sexual exposures

Transmission risk for a non-occupational exposure is more difficult to assess. However, any reported exposure should be managed as exposed, even if the exposure cannot be proven. There may be a need to counsel the exposed individual to decide whether taking PEP outweighs the risk of transmission. For example, in sexual exposures where the source individual (sexual partner) is known to have an undetectable HIV viral load, there is probably no need for HIV PEP.

### 3.4. Step 4: Assessing the source individual

An important part of determining the risk of HIV transmission is a thorough medical history of the source individual. This includes HIV status and HIV treatment history (if relevant) as well as the possible presence of any other infectious diseases. This may require blood tests to be done but awaiting these test results should not delay initiation of PEP in the exposed individual. If these details are not available a greater risk of HIV should be assumed.

*When the source individual is known*, every effort should be made to gain their voluntary, informed consent to perform the necessary laboratory investigations. This should be done as soon after the exposure as possible.^23^

*If the source individual is unknown*, uncontactable, unavailable for testing, or refuses consent, it should be the default to manage the exposed individual as if the source individual is HIV-positive. If the source individual is unable to give consent due to an altered level of consciousness or otherwise deemed unable to consent, national recommendations allowing testing in such circumstances should be followed.^24^ Testing of needles, sharps or other material involved in the exposure is not recommended as such investigations are unreliable.

The blood tests that should be performed on the source individual are shown in Table 3. If the source individual is found to test positive for any of these tests, post-test counselling and initiation of therapy or referral for further management is important.

**TABLE 3:** Investigations to be performed on blood from the source individual.

Infection/test	Laboratory test
HIV	4th-generation HIV ELISA (or qualitative HIV PCR in source < 2 years of age)
	HIV viral load‡
HBV	HBsAg
HCV	HCV Ab†
Syphilis	TP Ab/RPR

ELISA, enzyme-linked immunosorbent assay; HBV, hepatitis B virus; HBsAg, hepatitis B surface antigen; HCV, hepatitis C virus; HCV Ab, hepatitis C virus antibody; TP Ab, *Treponema pallidum* antibody; RPR, rapid plasma regain; PCR, polymerase chain reaction.

† Only if high risk for HCV and HCV-antibody status is unknown.

‡ Not routine, but useful if available.

If the source individual is known to be living with HIV and has a recent undetectable viral load (is stable on ART), PEP is usually not indicated unless the exposure was percutaneous (needlestick injury or physical assault). If the source individual is newly diagnosed with HIV, is not on ART or has a detectable viral load, PEP is indicated for all exposures with infectious fluid.

If there is suspicion of ART drug resistance in the source individual, an expert should be consulted regarding the appropriate PEP regimen. Alternatively, advice can be sought from other reliable sources (see Box 1).

BOX 1 Sources for seeking reliable information on PEP when the source individual has ART drug resistance.

**Table:** 

Reliable sources include:
UCT MIC HIV & TB Hotline: 0800 212 506 or UCT MIC HIV & TB Hotline: call 0800 212 506 or send an SMS or “Please Call Me” to 071 840 1572 or Download SA HIV & TB HCW Hotline App: https://mic.uct.ac.za/sa-hivtb-hotline-app
use SA HIV & TB HCW Hotline App: SA HIV & TB HCW Hotline App | Medicines Information Centre (uct.ac.za)
Right to Care HIV hotline: +27(0)82 352-6642

In cases of sexual assault, the law makes provision for HIV testing in alleged offenders.^24^ The victim, or an interested person, can apply for this to be done within 90 days of the alleged offence; however, this can be legally onerous and time consuming. *It is essential that this process does not delay initiating PEP.*

### 3.5. Step 5: Assessment of the exposed individual

Table 4 indicates routine investigations to be performed on blood from the exposed individual at the baseline assessment and at follow-up. It is important to know which investigations may be required in the case of occupational exposure and future compensation claims.

**TABLE 4:** Routine investigations to be performed on the exposed individual.

Test	Baseline	6 weeks	3 months
HIV	Rapid test plus 4th-generation HIV ELISA	4th-generation HIV ELISA	4th-generation HIV ELISA†
	If child < 2 years of age: qualitative HIV PCR	If child < 2 years of age: qualitative HIV PCR	If child < 2 years of age: qualitative HIV PCR
HBV	HBsAb‡	-	HBsAg‡
HCV	HCV Ab§	HCV PCR§	-
Syphilis	If sexual exposure or source positive for syphilis, TP Ab/RPR	-	TP Ab/RPR¶
Creatinine	If TDF part of PEP	-	-
FBC and differential¶	In child if zidovudine prescribed as PEP	-	-
Pregnancy (if appropriate)††	βHCG	βHCG	-

ELISA, enzyme-linked immunosorbent assay; HBV, hepatitis B virus; HBsAb, hepatitis B surface antibody; HBsAg, hepatitis B surface antigen; HCV, hepatitis C virus; HCV Ab, hepatitis C virus antibody; PCR, polymerase chain reaction; TP Ab, *Treponema pallidum* antibody; RPR, rapid plasma regain; FBC, full blood count; TDF, tenofovir disoproxil fumarate; PEP, post-exposure prophylaxis.

† 5th-generation ELISA may also be used.

‡ Can be omitted if exposed individual is known to be immune to HBV (through infection or vaccination).

§ Only if source is HCV Ab positive or unknown and at high risk of HCV infection.

¶ Repeat after 2 and 4 weeks in child on zidovudine.

†† In adolescents, this should be done if individual is Tanner stage 3 or greater.

#### 3.5.1. HIV testing

All exposed individuals should be offered counselling and HIV testing. A baseline HIV rapid test followed by a confirmatory HIV test should be performed and the results documented. Children > 2 years of age should be tested for HIV as per adult recommendations, while children < 2 years of age should have a qualitative HIV polymerase chain reaction (PCR) test done instead.

Follow-up testing for HIV should be undertaken 6 weeks and 3 months after the exposure event. Testing beyond 3 months post-exposure should be considered in the following situations:

- Indeterminate HIV test result at 3 months post-exposure.
- Ongoing high-risk behaviour or if requested by individual.
- A new exposure has been identified.

It is important to note that it is extremely rare to seroconvert while taking PEP.^12^ However, if the exposed individual tests HIV-positive at any point, they should be counselled, and linked to HIV treatment and care services as soon as possible.

### 3.6. Step 6: Initiating PEP in the exposed individual

It is essential that the exposed individual receive the appropriate PEP regimen as soon as possible after exposure and preferably as soon as possible on the same day that the initial assessment has been done. Compliance rates are higher when the full 28-day course is prescribed^25^ and, therefore, previously used ‘starter packs’ should not be used. Consideration should be made when selecting the appropriate regimen, keeping in mind potential side-effects, drug-drug interactions or other factors that may deem certain agents contraindicated. In the case of a child or adolescent, consideration to age and weight needs to be given and ART weight-based dosing charts should be consulted.^26^

#### 3.6.1. Selecting an appropriate ARV regimen

The use of three agents is recommended when providing PEP (see Table 5).^27^ For individuals ≥ 10 years and ≥ 30 kg, the preferred nucleoside/nucleotide reverse transcriptase inhibitor (NRTI) combination is tenofovir disoproxil fumarate (TDF) together with lamivudine (3TC) or emtricitabine (FTC) due to its tolerability, relatively few contra-indications and minimal monitoring requirements in short-term use. For those < 10 years or < 30 kg, the preferred backbone regimen is zidovudine (AZT) and lamivudine.

**TABLE 5:** Recommended PEP regimens.

Scenario	Recommended PEP regimen
**Individuals ≥ 10 years and ≥ 30 kg**	
First option	TDF (300 mg) + 3TC (300 mg) + DTG (50 mg) as a once-daily fixed-dose combination tablet (TLD). If on rifampicin† or carbamazepine, add additional DTG 50 mg once-daily (to be given within 12 h after TLD dose). For more information on drug-drug interactions refer to the SAHCS Adult ART 2023 guideline.^28^
If DTG contra-indicated	TDF (300 mg) + FTC (200 mg) + ATV/r (300/100 mg) as daily dose *or* TDF (300 mg) + FTC (200 mg) + LPV/r (200/50 mg) two tablets twice daily
If compromised renal function	If eGFR 10 mL/min – 50 mL/min: AZT 300 mg bd + 3TC 150 mg daily + DTG 50 mg daily If eGFR < 10 mL/min: AZT 300 mg daily + 3TC 50 mg daily + DTG 50 mg daily
**Individuals < 10 years or < 30 kg**	
Dose as per latest paediatric dosing chart^25^	
First option	Zidovudine (AZT) + 3TC + DTG
If DTG not available	Zidovudine (AZT) + 3TC + protease inhibitor (ATV/r or LPV/r)
Alternative option	Abacavir (ABC) + 3TC + DTG
	*ABC should only be used if there is no alternative* as there is a risk of a hypersensitivity reaction to ABC.

PEP, post-exposure prophylaxis; kg, kilograms; TLD, tenofovir/lamivudine/dolutegravir; AZT, zidovudine; LPV/r, lopinavir/ritonavir; TDF, tenofovir disoproxil fumarate; FTC, emtricitabine; ATV/r, atazanavir/ritonavir; eGFR, estimated glomerular filtration rate; DTG, dolutegravir; 3TC, lamivudine.

† Research currently underway to determine if additional dolutegravir is required in patients receiving concurrent rifampicin therapy. See the SAHCS Adult ART 2023 guideline.^28^

Dolutegravir (DTG) is the preferred third antiviral due to its excellent side-effect profile, rapid effect on HIV viral load, lower cost, and its availability as a fixed-dose combination with tenofovir and lamivudine (TLD). Other integrase inhibitors (e.g., bictegravir) may be considered.

For many years, a protease inhibitor (lopinavir/ritonavir or atazanavir/ritonavir) was routinely used as the third agent in first-line PEP despite poor tolerability and potential side-effects. This can still be used as a second choice if dolutegravir is unavailable or contra-indicated. If there is renal impairment, then zidovudine is an alternative, although it has side-effects.

If TLD as a fixed-dose combination is not available or appropriate, then it is important that the best possible combination be chosen for the exposed individual to minimise potential side-effects and the risk of early discontinuation, and drug-drug interactions.

#### 3.6.2. Counselling and support

**FIGURE 3:**
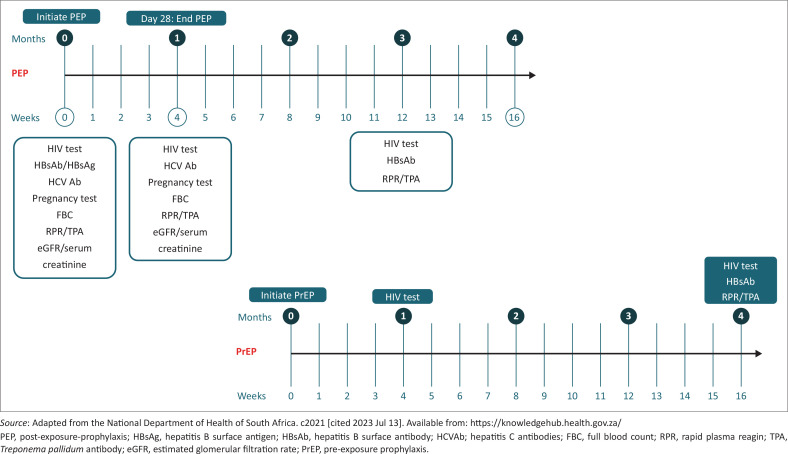
Post-exposure prophylaxis to PrEP transition.

Adequate and appropriate counselling is an integral part of the initial consultation with the exposed individual. Care should be taken to address specific concerns and key issues in a sensitive manner to minimise anxiety, and to ensure the effective use of PEP and the best possible outcome. Key counselling issues include:

- It is useful to inform the individual of commonly occurring side-effects of PEP medications (see Table 6) and to advise pre-emptive therapy to manage these symptoms, as well as symptoms that could indicate a more serious complication that should prompt the individual to seek immediate care. Most non-serious side-effects will resolve within 2 weeks.
- PEP studies report low completion rates amongst different population groups. A recent study among healthcare workers who have been exposed to HIV showed that there was a good initial uptake of ART (96%), but only 11% completed the full 28 day course.^29^ Uptake is improved by providing the full 28-days at the initial consult^25^ and emphasising daily adherence to the full 28-day course of treatment is important. Common daily activities can act as reminders, including setting a phone alarm or enlisting the help of a family member or friend.
- Considerable fear of acquiring HIV after an exposure is not uncommon, so it is important to address any concerns causing significant anxiety. Simple reassurance may not always be enough and additional interventions may be required. In addition, in traumatic exposures, like sexual assault, it may be difficult to adequately address all concerns due to the emotional state of the individual. In cases of significant anxiety and trauma, care should be taken to arrange urgent follow-up counselling or to refer to specific counselling services as soon as possible.
- PEP provision, in both occupational and non-occupational settings, is an ideal time to discuss interventions that could prevent potential further exposures. It is important to establish exposure risk and provide non-judgmental and practical advice to minimise further exposures. If appropriate, the healthcare provider should use the opportunity to discuss and encourage the use of PrEP, *particularly in cases of repeated PEP use or risk of regular potential HIV exposure*. See Figure 3 for a PEP to PrEP transition plan.
- It is also important to address secondary prevention. To reduce HIV transmission to others, exposed individuals should be counselled about utilising the following prevention measures during the first 3 months following an exposure:
  - use latex condoms and lubricant
  - avoid pregnancy and breastfeeding (if possible)
  - practise safe injection practices
  - avoid blood and tissue donations.

### 3.7. Step 7: Follow-up

Follow-up of the exposed individual, including monitoring for side-effects (see Table 6)^30^, is an important component of providing PEP. Follow-up investigations are indicated in Table 4.

**TABLE 6:** Common side-effects of PEP medications.

Medication	Side-effect
Tenofovir	Well tolerated Avoid if renal compromise (see Table 5)
Lamivudine & emtricitabine	Well tolerated
Dolutegravir	Well tolerated Uncommonly insomnia
Zidovudine	Gastrointestinal upset (nausea, vomiting), headache, fatigue, insomnia, metallic taste
Lopinavir/ritonavir	Gastrointestinal upset (nausea, vomiting, diarrhoea) is common

*Source:* Adapted from 2023 ART clinical guidelines for the management of HIV in adults, pregnancy and breastfeeding, adolescents, children, infants and neonates. Republic of South Africa National Department of Health; c2023 [cited 2023 Jul 13]. Available from: https://knowledgehub.health.gov.za

Modern ART regimens are very well tolerated but minor side-effects may occur with short-term use, such as in PEP. These can usually be treated effectively with reassurance and symptomatic treatment without the need to change or discontinue the current PEP regimen.

Exposed individuals with significant comorbidities (e.g., significant liver disease or renal disease) may require more monitoring with relevant investigations during PEP. However, healthy individuals do not require any additional investigations. If unsure, it is best to consult a specialist or reliable source (Box 1).

If an exposed individual tests positive for HIV at any time during the follow-up period, they should be linked to care as soon as possible and initiated on ART as per the latest HIV treatment guidelines.^28,30^

## 4. Other considerations

### 4.1. Hepatitis B

Hepatitis B virus (HBV) infection is relatively common in southern Africa and has a higher risk of transmission than HIV.^31^ Data from 2019 estimates that 6.7% of the South African population has chronic HBV infection.^31^ Tenofovir and lamivudine/emtricitabine are highly effective against HBV infection but there is some concern that, when used as part of a PEP regimen, stopping these agents may lead to an acute HBV flare; however, this is rare in practice. Screening and vaccination of all healthcare workers is strongly encouraged to prevent potential infection with HBV.

Management of exposure to HBV is outlined in Table 7.^31^ If an exposed individual is unsure of their HBV-antibody status (either by natural infection or vaccination), this should be investigated but this should not delay the initiation of HIV PEP.

**TABLE 7:** Management of HBV in the exposed individual.

Hepatitis B status of source individual	HBV vaccination status of exposed individual			
	Not vaccinated	Fully vaccinated, documented responder (HBsAb titre ≥ 10 mIU/mL)	Vaccinated, documented non-responder (HBsAb titre < 10 mIU/mL)	Vaccinated, HBsAb status unknown
HbsAg positive, or HbsAg unknown	Give HBIG, IM, 500 IU†	No action	Give HBIG, IM, 500 IU, then repeat after 1 month	Test exposed individual’s HBsAb**‡**:
	Initiate hepatitis B vaccine series		Re-initiate hepatitis B vaccine series (if not previously attempted)	If positive and titre ≥ 10 mIU/mL, no action
				If titre < 10 mIU/mL, give HBIG and hepatitis B vaccine booster, and recheck titre in one month
HbsAg negative	Initiate hepatitis B vaccine series	No action	Re-initiate hepatitis B vaccine series (if not previously been attempted)	Test exposed individual’s HBsAb:
				If positive and titre ≥ 10 mIU/mL, no action
				If < 10 mIU/mL, give hepatitis B vaccine booster and repeat titre after 1 month

*Source:* Adapted from National Guidelines for the Management of Viral Hepatitis. National Department of Health; c2019 [cited 2023 Jul 13]. Available from: https://knowledgehub.health.gov.za

HbsAg, hepatitis B surface antigen; HBIG, hepatitis B immunoglobulin; IM, intra-muscular; HBsAb, Hepatitis B surface antibodies.

† HBIG and first dose of vaccine should be administered simultaneously, at different sites.

‡ If testing for HBsAb will be delayed for > 24 h, treat as non-responder.

### 4.2. Hepatitis C

In South Africa, hepatitis C virus (HCV) is mostly found in certain risk groups including PWID, MSM, haemophiliacs or in certain high HCV prevalence settings, including some cities.^31^ Routine HCV testing in exposed individuals is not necessary, unless the source individual is known with HCV or is at high risk of HCV infection and their HCV-antibody (HCVAb) status is unknown. If the source individual tests HCVAb negative, no further testing is necessary for the exposed individual. However, where the source individual tests positive for HCVAb, the exposed individual should be tested for HCVAb at baseline and, if negative, have an HCV PCR done at 6 weeks follow-up after the exposure (see Table 4).

### 4.3. Sexually transmitted infections

Acquisition of STIs can occur with sexual exposure. If there is sexual exposure, it is recommended that the source individual be tested for syphilis using a rapid test (see Table 3) and, if positive, the exposed individual, should have a full clinical evaluation for signs or symptoms of syphilis infection and managed accordingly.^32^ Testing should be done at baseline and again at the 3-month follow-up visit (see Table 4). If the exposed individual is symptomatic for an STI, they should be managed as per the latest STI guidelines.^32^

Prophylaxis for STIs needs to be considered in children and adolescents who have survived sexual assault. All children with suspected penetrative injuries should receive STI prophylaxis. Details of recommended STI prophylaxis can be found in the South African National 2020 PEP guidelines.^20^

An STI in a child may be a presenting symptom of chronic sexual abuse, and it is important to document any STIs using standard microbiological tests as part of the criminal investigation.

### 4.4. Pregnancy

Exposed individuals of reproductive potential (including adolescents that are Tanner stage 3 or greater) in whom conception may be a risk should be offered pregnancy testing (see Table 4) and emergency contraception.

Should the individual be pregnant (of any gestation) or breastfeeding at the time of exposure, the preferred first-line PEP regimen remains TLD as it is known to be safe in such circumstances.^20,30,33^

### 4.5. Tetanus

Individuals who have experienced trauma associated with the exposure should be asked about their tetanus vaccination status and offered re-vaccination if appropriate.

### 4.6. Malaria

Routine testing of an exposed individual for malaria is not required unless the source individual is symptomatic for malarial infection. Consultation with an Infectious Disease expert is advised in such circumstances.
